# Rare Human Infection with Pacific Broad Tapeworm *Adenocephalus pacificus,* Australia

**DOI:** 10.3201/eid2208.160156

**Published:** 2016-08

**Authors:** Casey V. Moore, R.C. Andrew Thompson, Abdul Jabbar, John Williams, Kavita Rasiah, Louise Pallant, Ann P. Koehler, Caitlin Graham, Gerhard F. Weldhagen

**Affiliations:** SA Pathology, Adelaide, South Australia, Australia (C.V. Moore, G.F. Weldhagen);; Murdoch University, Murdoch, Western Australia, Australia (R.C.A. Thompson, L. Pallant);; University of Melbourne, Melbourne, Victoria, Australia (A. Jabbar);; Investigator Clinic, Port Lincoln, South Australia (J. Williams);; Women’s and Children’s Hospital, Adelaide (K. Rasiah);; SA Health Communicable Disease Control Branch, Adelaide (A.P. Koehler, C. Graham)

**Keywords:** Diphyllobothriosis, Adenocephalus pacificus, Australia, zoonoses, parasites, tapeworm, foodborne disease, Pacific broad tapeworm

**To the Editor**: Human diphyllobothriosis associated with the Pacific broad tapeworm *Adenocephalus pacificus* (syn. *Diphyllobothrium pacificum*) is a reemerging, global parasitic disease (*1*). Infection with the adult tapeworm occurs widely in piscivorous mammals, including humans, with various species of marine fish acting as intermediate hosts (*1*,*2*). In the Southern Hemisphere, the organism is well described in the coastal waters of South America, southern Africa, and Oceania (*2*). *A. pacificus* tapeworms have been recorded in pinnipeds in Australian territory as far back as 1923 (*3*). To our knowledge, no human case has been reported from this region to date.

A 3-year-old boy from a coastal town in South Australia’s Eyre Peninsula was brought to a medical clinic on July 29, 2015, with a 1-month history of poor appetite and loose bowel movements. His parents had noticed tapeworm-like organisms in his feces over a period of 2 days; the organisms had been discarded and were not available for examination. The child regularly ate raw marine fish, caught by his father in the Spencer Gulf during recreational fishing. The types of fish he consumed included southern bluefin tuna (*Thunnus maccoyii*), spotted sillago (*Sillaginodes punctatus*), and southern goatfish (*Upeneichthys vlamingii*). The child had never traveled outside Australia. Fecal and blood samples were collected for further analysis at SA Pathology (Adelaide, South Australia, Australia).

Macroscopic examination of a single, 6-cm portion of unfixed strobila without scolex, obtained from feces, revealed individual proglottids, wider than they were long, and a central rosette-shaped uterus in each proglottid. Further morphologic features could not be clearly visualized from the available clinical specimen. Microscopic examination of fecal sediment revealed unembryonated ellipsoidal eggs with an operculum and abopercular knob at opposite ends (*2*). The patient had been given a preliminary clinical diagnosis of diphyllobothriosis and received oral praziquantel (10 mg/kg) on 2 occasions, 12 days apart, without any complications. Blood test results, including hemoglobin level, erythrocyte volume, and vitamin B12 levels, were all within reference ranges. A follow-up stool sample collected 1 week after the first dose of praziquantel still exhibited operculated eggs; samples collected 3 weeks later did not contain any eggs.

We identified the tapeworm more specifically through molecular methods. Genomic DNA from segments preserved in 80% ethanol were extracted using a DNeasy Blood and Tissue Kit (QIAGEN, Valencia, CA, USA) following the manufacturer’s protocol. Four loci were PCR-amplified separately from the genomic DNA as described (*4*–*6*). The first locus (designated *cox1*) targeted the complete cytochrome *c* oxidase subunit 1 gene; the second locus (designated Dp*cox*1), located within the *cox1* gene, was amplified by using species-specific (*D. pacificum*) primers (*4*); the third locus (designated SSU) targeted the small subunit gene of RNA; and the fourth locus (designated ITS) targeted the first to second internal transcribed spacer regions. For each locus, automated DNA sequencing (BigDye Terminator v3.1 Kit; Applied Biosystems, Foster City, CA, USA) was performed by using the primers for PCR amplification (*4*–*6*) in separate reactions. We identified *cox*1 and Dp*cox*1 sequences by local alignment comparison (6 reading frames) using amino acid sequences conceptually translated online (http://www.ebi.ac.uk/Tools/st/emboss_transeq/) from respective genes of the reference sequences of *Diphyllobothrium* spp. available from GenBank. DNA sequencing results identified the tapeworm as *A. pacificus*.

Sequence data from this study have been submitted to GenBank (accession nos. KU519704–6). Morphologic voucher material is archived in the Australian Helmintological Collection, South Australian Museum (accession nos. AHC47709 and AHC36233).

Data obtained from *cox1* gene analysis (Figure, panel A) suggest that this isolate is indistinguishable from an *A. pacificus* tapeworm previously obtained from an Australian sea lion, *Neophoca cinerea* (GenBank accession no. KR269744), from Kangaroo Island and an Australian fur seal, *Arctocephalus pusillus* (GenBank accession no. KR269745), from Cape Woolamai in 1993 (*2*). Similarly, the human isolate we identified is indistinguishable from isolates previously obtained from fish and humans in Chile, Peru, and Spain (Figure, panel A). However, the human isolates from Spain (GenBank accession nos. HF969327 and KM520151) have been linked with fresh fish imported from South America (*7*,*8*). Small subunit and internal transcribed spacer sequence analysis showed that the human isolate was identical or closely related to isolates from Peru (Figure, panels B, C). This finding is in keeping with this species’ current predominant geographic distribution (*2*). 

Two recent food risk assessments in Australia did not recognize *A. pacificus* tapeworms as a potential zoonotic threat in marine finfish (*9*,*10*). Although our patient only consumed raw locally caught marine fish, thus acquiring a patent infection after accidental ingestion of plerocercoids, the fish species concerned have yet to be confirmed as suitable intermediate hosts of *A. pacificus* tapeworms (*1*). These findings, and reports spanning >90 years, suggest that *A. pacificus* tapeworms are endemic in piscivorous mammals off the Australian coast, and more human cases can be expected as pressure on marine fish stocks and consumption of uncooked fish increase. As a protective measure against this emerging foodborne zoonotic threat, the public should be made aware of risks associated with consumption of fresh, raw marine fish.

**Figure:**
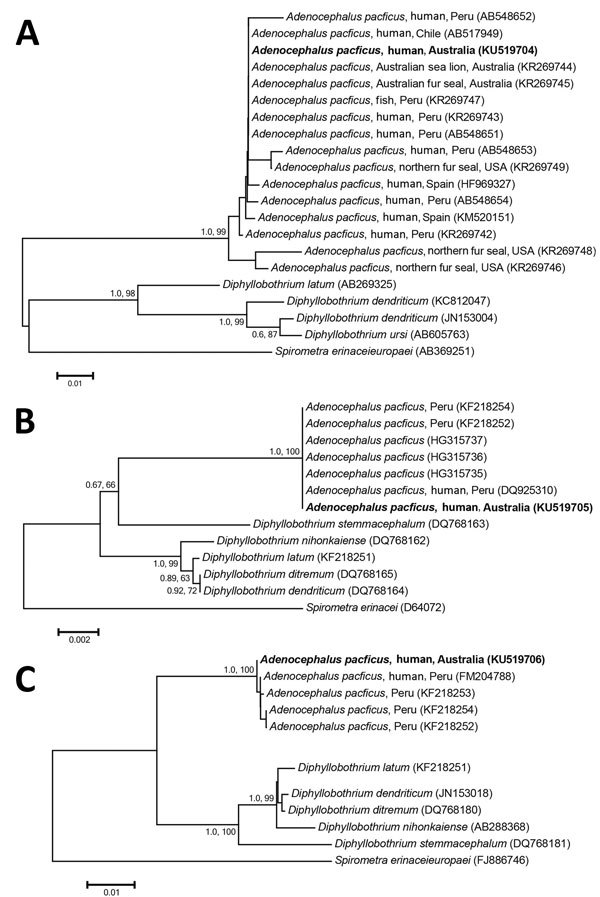
Phylogenetic relationship of genetic sequences from an *Adenocephalus pacificus* tapeworm obtained from a human in Australia (bold) and selected reference sequences. The relationship was inferred on the basis of DNA sequence analyses of cytochrome *c* oxidase 1 (A), small subunit RNA (B), and internal transcribed spacer (C) regions, using Bayesian inference and neighbor-joining phylogenetic methods. Topologies of trees generated by both methods were similar; thus, only neighbor-joining trees are shown here. *Spirometra* spp. were used as the outgroup. Nodal support is shown as posterior probability value (first) and bootstrap value (second) on the basis of 2 million generations for Bayesian inference and 10,000 replicates for neighbor joining, respectively. GenBank accession numbers are shown in parentheses. Scale bars indicate nucleotide substitutions per site.
